# The survival impact of palliative radiotherapy on synchronous metastatic pancreatic ductal adenocarcinoma: metastatic site can serve for radiotherapy-decision

**DOI:** 10.7150/jca.64800

**Published:** 2022-01-01

**Authors:** Biaoxiang Xu, Yuan Zhou, Qian Pei, Fengbo Tan, Lilan Zhao, Cenap Güngör, Dan Wang, Yuqiang Li, Wenxue Liu, Zhongyi Zhou

**Affiliations:** 1 Department of General Surgery, Xiangya Hospital, Central South University, Changsha, China.; 2 National Clinical Research Center for Geriatric Disorders, Xiangya Hospital, Central South University, Changsha, China.; 3 Department of Thoracic surgery, Fujian Provincial Hospital, Fuzhou, China.; 4 Department of General Visceral and Thoracic Surgery, University Medical Center Hamburg-Eppendorf, Hamburg, Germany.; 5 Department of Cardiology, Xiangya Hospital, Central South University, Changsha, China.; 6 Department of Rheumatology, Guangdong Provincial People's Hospital, Guangdong Academy of Medical Sciences, Guangzhou, China.

**Keywords:** PDAC, radiotherapy, metastatic site, overall survival, SEER database

## Abstract

**Background:** The metastatic site seems to represent a malignancy with a different biological characteristic and is an important prognostic factor in metastatic pancreatic ductal adenocarcinoma (mPDAC). Palliative radiotherapy is a therapeutic option, and usually used for pain management in the treatment of mPDAC. The real-world effect of radiotherapy on the survival outcomes of mPDAC patients might do exist and is worth exploring.

**Methods:** Data from the Surveillance, Epidemiology, and End Results (SEER) was extracted to identify mPDAC diagnosed in the periods of 2010-2016. The statistical methods included Pearson's chi-square test, Log-rank test, Cox regression model and propensity score matching (PSM).

**Results:** Radiotherapy was able to improve the overall survival of PDAC with liver metastasis (p<0.001), but not for PDAC patients with lung (p=0.130), bone (p=0.451) and brain metastasis (p=0.226) before PSM. Radiotherapy can only a prognostic factor for PDAC liver metastasis (p=0.001) in the cox regression analysis. The survival curves provided consistent results with cox regression analysis (PDAC with liver metastasis: p=0.023, PDAC with lung metastasis: p=0.528, PDAC with bone metastasis: p=0.210, PDAC with brain metastasis: p=0.106) after PSM. We continue to divided PDAC liver patients into PDAC-liver-metastasis with and without lung, bone, and/or brain (LBB) metastasis. Finally, radiotherapy can be used as a feasible treatment to prolong the overall survival of patients with PDAC liver metastasis without LBB metastasis.

**Conclusions:** Radiotherapy can be used as a feasible treatment to prolong the overall survival of patients with PDAC liver metastasis without LBB metastasis.

## Introduction

Pancreatic ductal adenocarcinoma (PDAC) is currently the 4^th^ most frequent cause of cancer-related death due to its high aggressiveness, early metastatic spread and pronounced resistance to therapy 1, and projected to become the second most lethal tumor by the year 2030 2. The majority of patients with PDAC exhibited metastasis at the time of diagnosis due to the lack of effective early diagnostic markers 3. The 5-year overall survival (OS) for metastatic pancreatic cancer remains at 2%, with a median life expectancy of < 1 year with current treatments 4.

A previous study reported that the metastatic site is an important prognostic factor in mPDAC 5. More important, the metastatic site seems to represent a malignancy with a different biological characteristics 6. Palliative radiotherapy is a therapeutic option, and usually used for pain management in the treatment of mPDAC 4. However, the effect of palliative radiotherapy on survival of mPDAC is still unclear. The real-world effect of radiotherapy on the survival outcomes of mPDAC patients might do exist and is worth exploring.

This study herein took advantage of the large patient population of the Surveillance, Epidemiology, and End Results (SEER) database to comprehensively examine the impact of radiotherapy on survival outcomes of mPDAC based on the metastatic site. These data can inform pancreatic oncologists in counseling patients with stage IV PDAC with synchronous metastatic disease seeking prognostic information when weighing radiotherapy-decision.

## Materials and Methods

### Patients Screening

**Figure 1:**
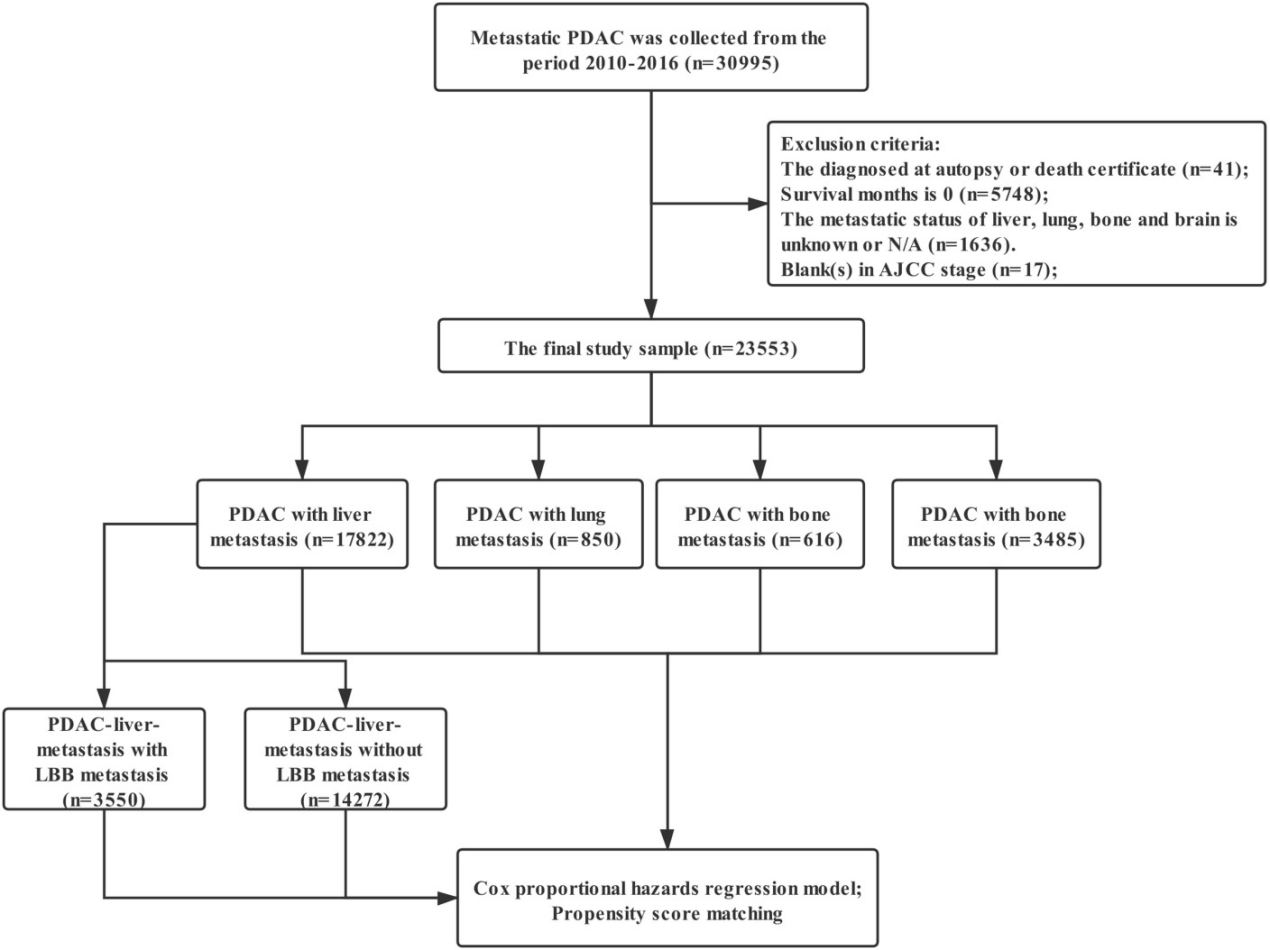
** The flow diagram.** Inclusion criteria: The PDAC patients (Histology recode: 8140-8389, 8440-8499) with stage M1 was collected from the period 2010-2016, 30,995 patients in total. Exclusion criteria: the diagnosed at autopsy or death certificate (n=41); Survival months is 0 (n=5748); The metastatic status of liver, lung, bone and brain is unknown or N/A (n=1636); blank(s) in AJCC stage (n=17); The final study sample contained 23,553 patients.

Data were extracted from the SEER linked database in this retrospective analysis. The SEER Program of the National Cancer Institute (https://seer.cancer.gov/) is an authoritative source of information on cancer incidence and survival in the United States (U.S.) that is updated annually. The PDAC patients (Histology recode: 8140-8389, 8440-8499) with stage M1 was collected from the period 2010-2016, 30,995 patients in total. Exclusion criteria: the diagnosed at autopsy or death certificate (n=41); Survival months is 0 (n=5748); The metastatic status of liver, lung, bone and brain is unknown or N/A (n=1636); blank(s) in AJCC stage (n=17); The final study sample contained 23,553 patients (Figure 1). For each patient, the following data was acquired: insurance, age at diagnosis, marital status, gender, race, primary tumor location, grade, histological type, T stage, N stage, surgery for primary tumor, metastatic site, radiotherapy and chemotherapy.

### Statistical Analysis

The statistical methods were performed as previously described 7. Intergroup comparisons were analyzed using Pearson's chi-square test. Log-rank test was used to compare overall survival (OS) among different groups. A hazard ratio (HR) and a 95% confidence interval (CI) were evaluated by a univariate and multivariate Cox proportional hazards regression model. Univariate analysis of variables with a *p-*value lower than 0.05 or the *p*-value of radiotherapy were included in the Cox regression model for multivariate analysis. In order to eliminate the influence of other variables, we conducted a propensity score matching (PSM). Statistical analyses were performed with IBM SPSS statistics trial ver. 25.0 (IBM, Armonk, NY, USA). All reported *p*-values lower than 0.05 were considered significant.

## Results

The characteristics of patients with mPDAC enrolled from the SEER database were summarized in Table 1. The total population included 17822 (75.67%) cases of liver metastasis, 4717 (20.03%) patients with lung metastasis, 1666 (7.07%) ones with bone metastasis as well as 159 (0.68%) PDAC brain metastasis patients. 1379 (5.85%) mPDAC patients received radiotherapy. There was no difference in seven variables such as insurance, gender, marital status, race, histologic type, T and N staging between the radiotherapy group and the non-radiotherapy group. The proportion of lung, bone and brain metastases in the radiotherapy group was significantly higher than that in the non-radiotherapy group, while the rate of patients with liver metastases in the radiotherapy cohort was obviously lower than that in the non-radiotherapy group.

**Figure 2:**
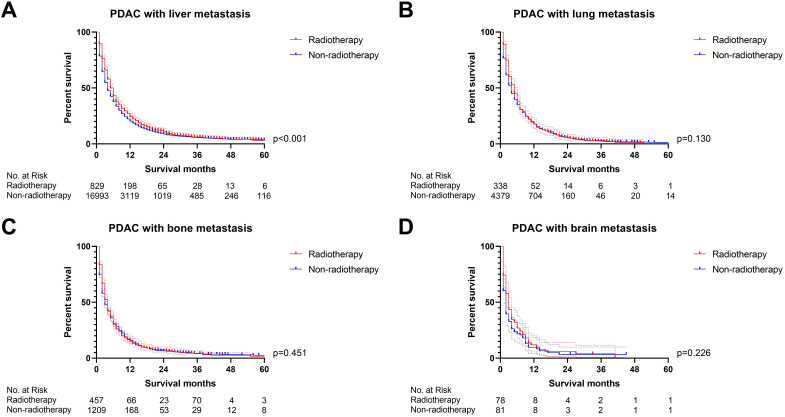
The survival curves showed that **(A)** radiotherapy was able to improve OS of PDAC with liver metastasis before PSM (p<0.001); **(B)** PDAC with lung metastasis (p=0.130), **(C)** PDAC with bone metastasis (p=0.451) and **(D)** PDAC with brain metastasis (p=0.226) cannot obtain survival benefit from radiotherapy before PSM.

**Figure 3:**
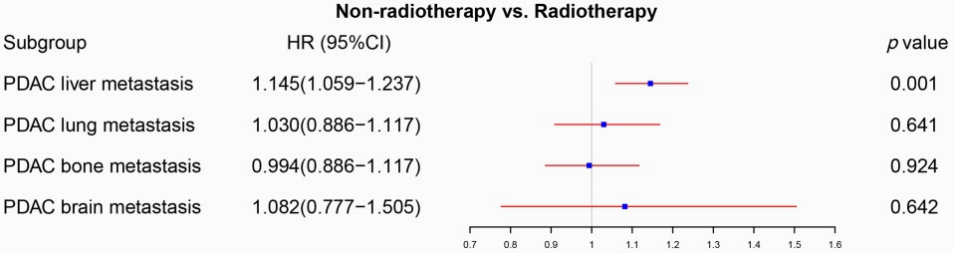
The forest plot was used to display the role of radiotherapy in the multivariable Cox regression. Radiotherapy can be used as a prognostic factor for PDAC with liver metastasis (p=0.001), but not for PDAC with lung metastasis (p=0.641), PDAC with bone metastasis (p=0.924) and PDAC with brain metastasis (p=0.642). (The results were extracted from Table S1).

**Figure 4:**
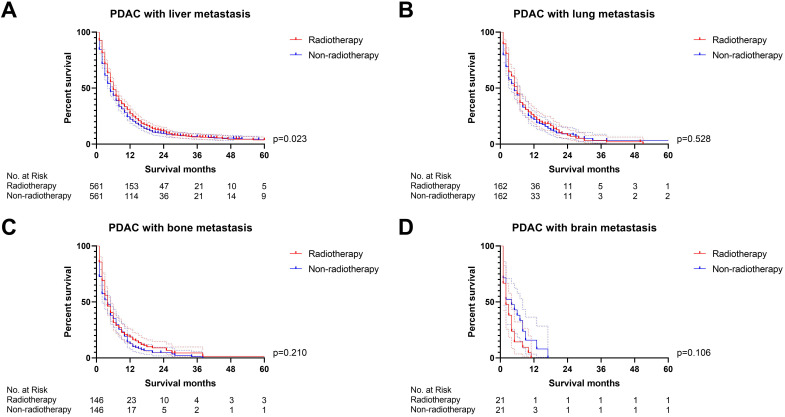
The survival curves demonstrated that **(A)** PDAC with liver metastasis can obtain survival benefit from radiotherapy after PSM (p=0.023); **(B-D)** radiotherapy was not able to improve OS of PDAC with lung (p=0.528), bone (p=0.210) and brain metastasis (p=0.106) after PSM (the results of PSM were summarized in Table S2).

Firstly, we compared the survival difference between the radiotherapy and non-radiotherapy cohort in the PDAC patients with liver, lung, bone and brain metastasis respectively. Radiotherapy was able to improve the overall survival of PDAC with liver metastasis (p<0.001), but not for PDAC patients with lung (p=0.130), bone (p=0.451) and brain metastasis (p=0.226) (Figure 2). Multivariate Cox proportional hazards regression model was then utilized to adjust the influence of other variables. Univariate Cox regression analysis of variables with a *p-*value lower than 0.05 or the *p*-value of radiotherapy were included in the multivariate analysis (Table S1), which confirmed that radiotherapy can only a prognostic factor for PDAC liver metastasis (p=0.001, Figure 3). We also conducted propensity score matching (PSM) (Table S2) to eliminate the influence of other variables and got consistent results with cox regression analysis (PDAC with liver metastasis: p=0.023, PDAC with lung metastasis: p=0.528, PDAC with bone metastasis: p=0.210, PDAC with brain metastasis: p=0.106; Figure 4).

**Figure 5:**
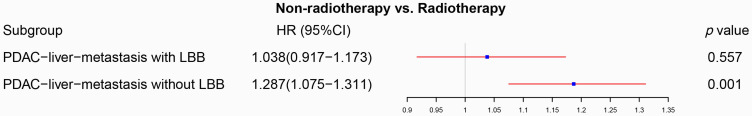
The forest plot illustrated that radiotherapy was not able to significantly affect OS of PDAC-liver-metastasis with LBB (p=0.557), and can be used as a prognostic factor for PDAC-liver-metastasis without LBB (p=0.001) (the results were extracted from Table S3).

**Figure 6:**
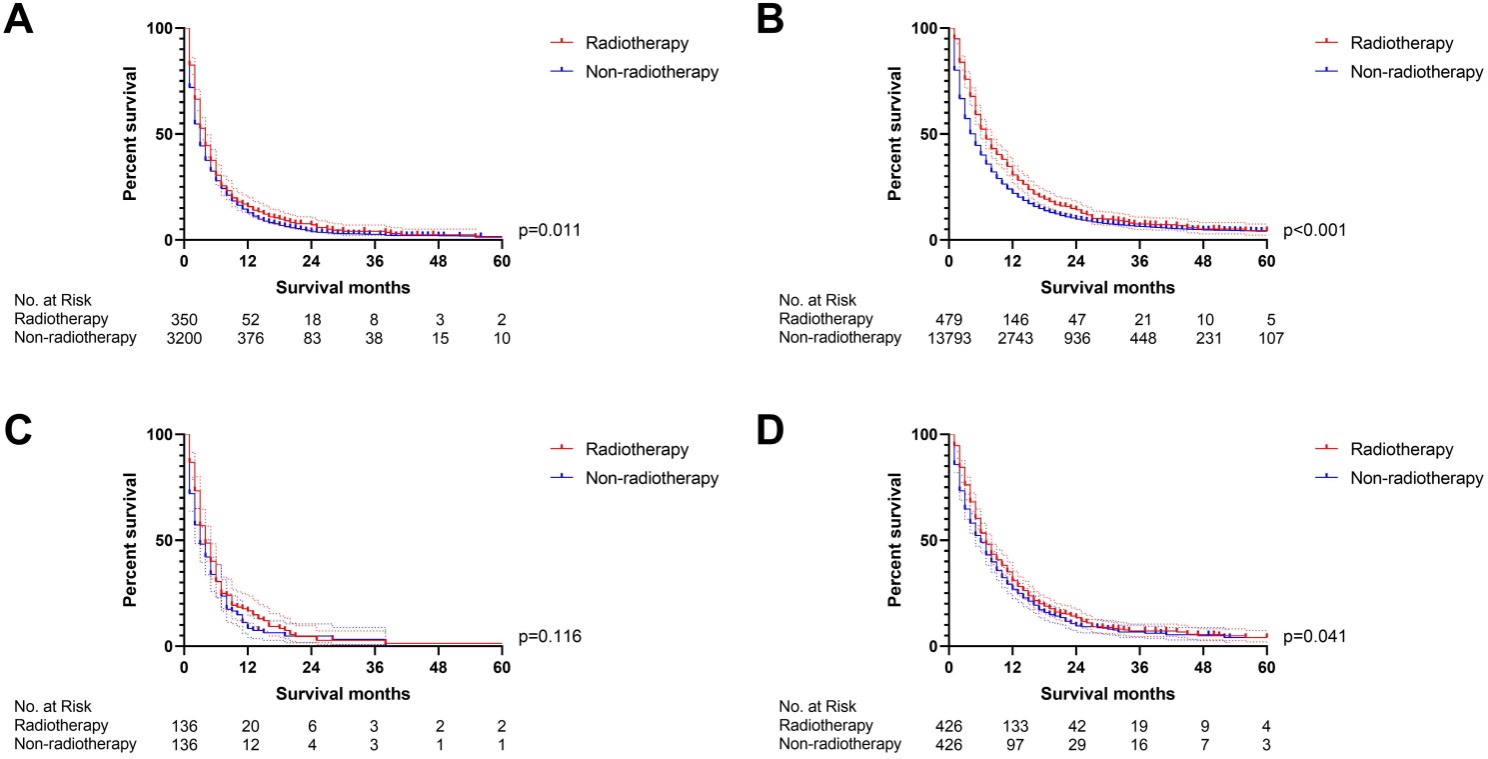
The survival curves indicated that **(A)** PDAC-liver-metastasis with LBB (p=0.011) and **(B)** PDAC-liver-metastasis without LBB (p<0.001) can obtain survival benefit from radiotherapy before PSM; However, radiotherapy cannot provide survival benefit for** (C)** PDAC-liver-metastasis with LBB (p=0.116) after PSM; **(D)** radiotherapy was able to improve OS of PDAC-liver-metastasis without LBB (p=0.041) after PSM (the results of PSM were summarized in Table S4).

However, can patients with PDAC liver metastasis combined with lung, bone, and/or brain (LBB) metastasis benefit from radiotherapy? We continue to divided PDAC liver patients into PDAC-liver-metastasis with and without LBB metastasis. The univariate Cox regression analysis displayed that radiotherapy was associated with overall survival in both of PDAC-liver-metastasis with and without LBB metastasis (Table S3), while the multivariate Cox proportional hazards regression model confirmed that PDAC-liver-metastasis with LBB metastasis cannot obtain survival benefit from radiotherapy (p=0.557, Figure 5). A propensity score matching (PSM) (Table S4) was conducted to further verify the results of Cox regression models. Radiotherapy was able to provide survival benefit to both of PDAC-liver-metastasis with (p=0.011, Figure 6A) and without LBB metastasis (p<0.001, Figure 6B) before PSM. Nevertheless, PDAC-liver-metastasis with LBB metastasis failed to get survival benefit from radiotherapy (p=0.116, Figure 6C), which significantly improve overall survival of PDAC-liver-metastasis without LBB metastasis (p=0.041, Figure 6D) after PSM. Collectively, radiotherapy can be used as a feasible treatment to prolong the overall survival of patients with PDAC liver metastasis without LBB metastasis.

## Discussion

The primary goal of treatment for metastatic pancreatic cancer is to relieve symptoms and prolong the survival of patients 8. Pancreatic scholars have been committed to improving the survival of PDAC in the past several decades. Several surgical concepts including total mesopancreatic excision (TMpE) and accurate assessment of the resection margins have been identified as an important factor improving the survival of patients with PDAC 9. Promising chemotherapy regimens, including nab-paclitaxel plus gemcitabine and FOLFIRINOX, also demonstrated superiority for PDAC patients 10-12. Use of RT might be associated with a favorable clinical outcome in patients with locally-advanced and metastatic pancreatic cancer 13. Meanwhile, the efficacy of radiotherapy become satisfactory, with low secondary damage due to technological advancement 14, 15. Especially, intraoperative radiotherapy (IORT) can be used for the purpose of preventing local recurrence after curative resection or as pain control for patients with an unresectable tumor 16. Our previous study also explored the role of radiotherapy and confirmed that radiotherapy was able to improve survival for locoregional PDAC 17. However, the advances in treatments contributed little to prolong the survival of mPDAC. In fact, patients with mPDAC are usually recommended to receive chemotherapy and radiation therapy is only suggested to control and relieve pain caused by primary tumor compressing nerves or the spine 18, 19. It is necessary to explore the survival impact of palliative radiotherapy on synchronous mPDAC.

To the best of our knowledge, this study was the first study to specifically investigate the survival effect of radiotherapy on metastatic PDAC patients based on the metastatic site. In fact, some recent studies reported that patients with disseminated disease need to accept immediate palliative short RT, even those in an oligometastasized stage should receive fractionated RT or radiosurgery 20. However, it is unclear whether the metastatic sites can serve for radiotherapy-decision in patients with synchronous metastatic PDAC. The specific metastatic sites may reflect the molecular background and clinicopathological characteristics of pancreatic cancer subtypes 21-25. However, there has been limited consensus on whether metastatic patterns are correlated with different prognosis and treatment efficacy in pancreatic cancer 26. This study taking advantage of the large patient population of the SEER database is able to provide credible evidence regarding radiotherapy options and promote individualized treatment for mPDAC. More important, the metastatic site may be used as a reference factor in radiotherapy-decision making for mPDAC.

A pilot research confirmed that the combination of immune checkpoint inhibitors and radiotherapy was an acceptable safety profile and a modest survival benefit in patients with metastatic PDAC 27. Another study analyzing the SEER database reported that radiotherapy can be used as a prognostic factor for mPDAC 28. Regrettably, these studies ignored the influence of the metastatic site in mPDAC. Recent research investigating the impact of different metastatic patterns on survival confirmed that the metastatic site is an important prognostic factor in mPDAC 5. Furthermore, a survival nomogram calculating risk scores of all prognostic factors for mPDAC demonstrated that PDAC with liver metastasis contributed most to survival comparing to that with other sites metastasis 28. Our study innovatively explored the role of radiotherapy on PDAC with different metastatic sites and suggested that radiotherapy was able to improve the overall survival of patients with PDAC liver metastasis without LBB metastasis. The metastatic site may be determined by the molecular phenotype, which is related to the sensitivity of radiotherapy 29-31. Moreover, we also found that chemotherapy provided inconsistent survival benefits to PDAC with different metastatic sites (Table S1, Table S3 and Figure S1), indicating that the metastatic site may also associate with the chemotherapeutic sensitivity. Collectively, the credible evidence based on the large patient population of the SEER database can provide a new treatment strategy and promote individualized treatment for mPDAC.

Unfortunately, the radiotherapy rate in PDAC patients with liver metastasis (829/17822, 4.65%) was disappointingly low comparing with that with lung (338/4717, 7.18%), bone (457/1666, 27.43%) and brain metastasis (78/159, 49.06%). Actually, only 3.36% (479/14272) of patients with PDAC liver metastasis without LBB metastasis received radiotherapy in this study. Therefore, it is absolutely inadequate regarding the usage rate of radiotherapy in mPDAC. Moreover, the inconsistent survival effect of radiotherapy among PDAC with different metastatic sites also suggested that oncologists should investigate the most effective chemotherapy regimen suitable for specific metastasis sites. More important, it is necessary to explore the molecular mechanism of the different metastatic patterns of PDAC, which is helpful for clinicians to predict the most likely metastatic sites of pancreatic cancer, so as to formulate targeted treatment strategies.

Limitations of this study include: (1) the use of retrospective data; (2) we failed to analysis mPDAC with distant lymph node metastasis since the SEER database only recorded four sites of metastasis at diagnosis. (3) the SEER database provides limited information on treatment regimens, including details of adjuvant chemotherapy and surgery on metastasis. (4) Due to SEER database does not provide detailed data on the number of metastatic organs in PDAC patients with metastatic disease, this study could not conclude whether palliative radiotherapy could improve the prognosis of PDAC patients with lung-limited, bone-limited or brain-limited. (5) We failed to validate our findings using data from our institution due to lacking of enough sample.

## Conclusion

The metastatic site can serve for radiotherapy-decision in patients with synchronous metastatic PDAC. Radiotherapy can be used as a feasible treatment to prolong the overall survival of patients with PDAC liver metastasis without LBB metastasis.

## Supplementary Material

Supplementary figure and tables.

## Figures and Tables

**Table 1:** Characteristics of metastatic PDAC

Characteristics	Total (n=23553)		Non-radiotherapy (n=22174)		Radiotherapy (n=1379)		*p*-value
	N	%	N	%	N	%	
**Insurance**							0.245
Yes	22569	95.82%	21256	95.86%	1313	95.21%	
No/NOS	984	4.18%	918	4.14%	66	4.79%	
**Gender**							0.135
Female	11014	46.76%	10396	46.88%	618	44.82%	
Male	11539	48.99%	10778	48.61%	761	55.18%	
**Age (years)**							<0.001
<65	9649	40.97%	8985	40.52%	664	48.15%	
≥65	13904	59.03%	13189	59.48%	715	51.85%	
**Marital status**							0.556
Married	13382	56.82%	12588	56.77%	794	57.58%	
Unmarried/NOS	10171	43.18%	9586	43.23%	585	42.42%	
**Race**							0.533
White	18701	79.40%	17597	79.36%	1104	80.06%	
Non-white	4852	20.60%	4577	20.64%	275	19.94%	
**Primary tumor location**							0.015
Pancreas Head	8532	36.22%	7977	35.97%	555	40.25%	
Pancreas Body/Tail	8676	36.84%	8210	37.03%	466	33.79%	
Pancreas Other	6345	26.94%	5987	27.00%	358	25.96%	
**Pathologic grade**							0.004
Grade I/II	2635	11.19%	2465	11.12%	170	12.33%	
Grade III/IV	2455	10.42%	2274	10.26%	181	13.13%	
Unknown	18463	78.39%	17435	78.63%	1028	74.55%	
**Histologic type**							0.082
Adenocarcinomas	22478	95.44%	21175	95.49%	1303	94.49%	
SRCC/MCC	1075	4.56%	999	4.51%	76	5.51%	
**T staging**							0.612
T0-3	13616	57.81%	12850	57.95%	766	55.55%	
T4	4517	19.18%	4175	18.83%	342	24.80%	
Tx	5420	23.01%	5149	23.22%	271	19.65%	
**N staging**							0.464
N0	12028	51.07%	11369	51.27%	659	47.79%	
N+	7889	33.49%	7356	33.17%	533	38.65%	
Nx	3636	15.44%	3449	15.55%	187	13.56%	
**Pancreatectomy**							<0.001
Yes	756	3.21%	687	3.10%	69	5.00%	
No	22797	96.79%	21487	96.90%	1310	95.00%	
**Chemotherapy**							<0.001
Yes	14696	62.40%	13671	61.65%	1025	74.33%	
No	8857	37.60%	8503	38.35%	354	25.67%	
**Bone metastasis**							<0.001
Yes	1666	7.07%	1209	5.45%	457	33.14%	
No	21887	92.93%	20965	94.55%	922	66.86%	
**Brain metastasis**							<0.001
Yes	159	0.68%	81	0.37%	78	5.66%	
No	23394	99.32%	22093	99.63%	1301	94.34%	
**Liver metastasis**							<0.001
Yes	17822	75.67%	16993	76.63%	829	60.12%	
No	5731	24.33%	5181	23.37%	550	39.88%	
**Lung metastasis**							<0.001
Yes	4717	20.03%	4379	19.75%	338	24.51%	
No	18836	79.97%	17795	80.25%	1041	75.49%	

SRCC: Signet ring cell carcinoma; MCC: Mucinous cell carcinoma.

## References

[1] Siegel RL, Miller KD, Jemal A Cancer statistics, 2018. 2018; 68: 7-30.

[2] Rahib L, Smith BD, Aizenberg R, Rosenzweig AB, Fleshman JM, Matrisian LM (2014). Projecting cancer incidence and deaths to 2030: the unexpected burden of thyroid, liver, and pancreas cancers in the United States. Cancer research.

[3] Lianyuan T, Deyu L, Haibo Y, Yadong D, Guanjing T (2021). Clinical features and prognostic factors of elderly patients with metastatic pancreatic cancer: a population-based study. Aging.

[4] Sohal DP, Mangu PB, Khorana AA, Shah MA, Philip PA, O'Reilly EM (2016). Metastatic Pancreatic Cancer: American Society of Clinical Oncology Clinical Practice Guideline. Journal of clinical oncology: official journal of the American Society of Clinical Oncology.

[5] He C, Huang X, Zhang Y, Lin X, Li S (2021). The impact of different metastatic patterns on survival in patients with pancreatic cancer. Pancreatology: official journal of the International Association of Pancreatology.

[6] Cavallaro P, Bordeianou L, Stafford C, Clark J, Berger D, Cusack J (2020). Impact of Single-organ Metastasis to the Liver or Lung and Genetic Mutation Status on Prognosis in Stage IV Colorectal Cancer. Clinical colorectal cancer.

[7] Li Y, Zhao L, Gungor C, Tan F, Zhou Z, Li C (2019). The main contributor to the upswing of survival in locally advanced colorectal cancer: an analysis of the SEER database. Therapeutic advances in gastroenterology.

[8] NCCN (2019). NCCN Clinical Practice Guidelines in Oncology (NCCN Guidelines). Pancreatic Adenocarcinoma (Version 2. 2019).

[9] Li Y, Liu W, Zhao L, Xu Y, Yan T, Yang Q (2020). The Main Bottleneck for Non-Metastatic Pancreatic Adenocarcinoma in Past Decades: A Population-Based Analysis. Medical science monitor: international medical journal of experimental and clinical research.

[10] Conroy T, Desseigne F, Ychou M, Bouche O, Guimbaud R, Becouarn Y (2011). FOLFIRINOX versus gemcitabine for metastatic pancreatic cancer. The New England journal of medicine.

[11] Silvestris N, Brunetti O, Vasile E, Cellini F, Cataldo I, Pusceddu V (2017). Multimodal treatment of resectable pancreatic ductal adenocarcinoma. Critical reviews in oncology/hematology.

[12] Silvestris N, Longo V, Cellini F, Reni M, Bittoni A, Cataldo I (2016). Neoadjuvant multimodal treatment of pancreatic ductal adenocarcinoma. Critical reviews in oncology/hematology.

[13] Yang SH, Guo JC, Yeh KH, Tien YW, Cheng AL, Kuo SH (2016). Association of radiotherapy with favorable prognosis in daily clinical practice for treatment of locally advanced and metastatic pancreatic cancer. Journal of gastroenterology and hepatology.

[14] Ben-Josef E, Shields AF, Vaishampayan U, Vaitkevicius V, El-Rayes BF, McDermott P (2004). Intensity-modulated radiotherapy (IMRT) and concurrent capecitabine for pancreatic cancer. International journal of radiation oncology, biology, physics.

[15] Safran H, Moore T, Iannitti D, Dipetrillo T, Akerman P, Cioffi W (2001). Paclitaxel and concurrent radiation for locally advanced pancreatic cancer. International journal of radiation oncology, biology, physics.

[16] Nagai S, Fujii T, Kodera Y, Kanda M, Sahin TT, Kanzaki A (2011). Prognostic implications of intraoperative radiotherapy for unresectable pancreatic cancer. Pancreatology: official journal of the International Association of Pancreatology.

[17] Wang D, Liu C, Zhou Y, Yan T, Li C, Yang Q (2020). Effect of neoadjuvant radiotherapy on survival of non-metastatic pancreatic ductal adenocarcinoma: a SEER database analysis. Radiation oncology (London, England).

[18] Lambert A, Schwarz L, Borbath I, Henry A, Van Laethem JL, Malka D (2019). An update on treatment options for pancreatic adenocarcinoma. Therapeutic advances in medical oncology.

[19] Lahoud MJ, Kourie HR, Antoun J, El Osta L, Ghosn M (2016). Road map for pain management in pancreatic cancer: A review. World journal of gastrointestinal oncology.

[20] Habermehl D, Brecht IC, Debus J, Combs SE (2014). Palliative radiation therapy in patients with metastasized pancreatic cancer - description of a rare patient group. European journal of medical research.

[21] Lovecek M, Skalicky P, Chudacek J, Szkorupa M, Svebisova H, Lemstrova R (2017). Different clinical presentations of metachronous pulmonary metastases after resection of pancreatic ductal adenocarcinoma: Retrospective study and review of the literature. World journal of gastroenterology.

[22] Matsuki R, Sugiyama M, Takei H, Kondo H, Fujiwara M, Shibahara J (2018). Long-term survival with repeat resection for lung oligometastasis from pancreatic ductal adenocarcinoma: a case report. Surgical case reports.

[23] Reichert M, Bakir B, Moreira L, Pitarresi JR, Feldmann K, Simon L (2018). Regulation of Epithelial Plasticity Determines Metastatic Organotropism in Pancreatic Cancer. Developmental cell.

[24] Crawford HC, Pasca di Magliano M, Banerjee S (2019). Signaling Networks That Control Cellular Plasticity in Pancreatic Tumorigenesis, Progression, and Metastasis. Gastroenterology.

[25] Wu L, Zhu L, Xu K, Zhou S, Zhou Y, Zhang T (2021). Clinical significance of site-specific metastases in pancreatic cancer: a study based on both clinical trial and real-world data. Journal of Cancer.

[26] Adamska A, Falasca M (2018). Epithelial plasticity is crucial for pancreatic cancer metastatic organotropism. Annals of translational medicine.

[27] Xie C, Duffy AG, Brar G, Fioravanti S, Mabry-Hrones D, Walker M (2020). Immune Checkpoint Blockade in Combination with Stereotactic Body Radiotherapy in Patients with Metastatic Pancreatic Ductal Adenocarcinoma. Clinical cancer research: an official journal of the American Association for Cancer Research.

[28] Lu Y, Zhou Y, Cao Y, Shi Z, Meng Q (2019). A Competing-Risks Nomogram in Patients with Metastatic Pancreatic Duct Adenocarcinoma. Medical science monitor: international medical journal of experimental and clinical research.

[29] Osipov A, Blair AB, Liberto J, Wang J, Li K, Herbst B Inhibition of focal adhesion kinase enhances antitumor response of radiation therapy in pancreatic cancer through CD8+ T cells. 2021; 18: 206-14.

[30] Mohamed AA, Thomsen A, Follo M, Zamboglou C, Bronsert P, Mostafa H FAK inhibition radiosensitizes pancreatic ductal adenocarcinoma cells *in vitro*. 2021; 197: 27-38.

[31] Zhu X, Cao Y, Ju X, Zhao X, Jiang L, Ye Y (2021). Personalized designs of adjuvant radiotherapy for pancreatic cancer based on molecular profiles. Cancer science.

