# Supplementary material for: The survival impact of palliative radiotherapy on synchronous metastatic pancreatic ductal adenocarcinoma: metastatic site can serve for radiotherapy-decision
Source: J Cancer. 2022 Jan 1;13(2):385–92. doi: 10.7150/jca.64800 (PMC8771529; doi:10.7150/jca.64800)
Supplement: Supplementary file 1 — Supplementary figure and tables. [file jcav13p0385s1.pdf]

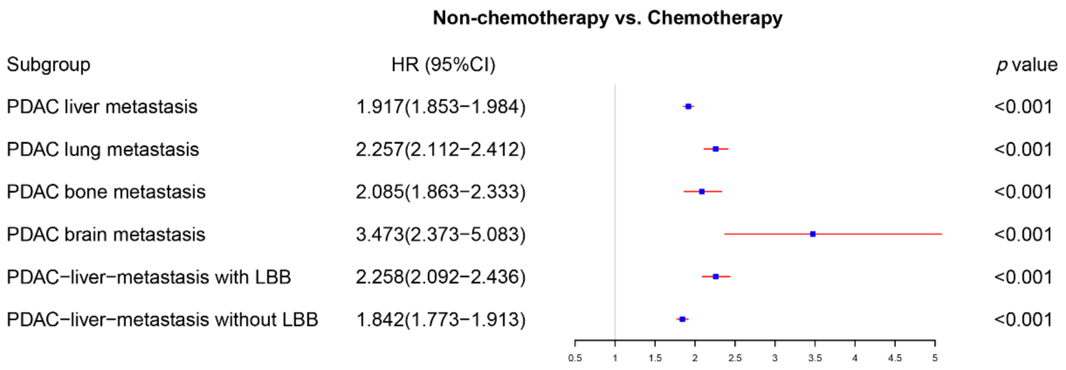

Figure S1: The forest plot illustrated that chemotherapy provided inconsistent survival

Table S1 Univariable and multivariable Cox regression model in mPDAC patients

PDAC liver metastasis								
Characteristics	Univariable analysis				Multivariable analysis			
	HR	95% CI lower	95% CI upper	*p*-value	HR	95% CI lower	95% CI upper	*p*-value
**Radiotherapy**				**<0.001**				**0.001**
**Yes**		**reference**				**reference**		
**No**	**1.151**	**1.069**	**1.241**	**<0.001**	**1.145**	**1.059**	**1.237**	**0.001**
Insurance				0.007				0.006
Yes		reference				reference		
No/NOS	1.111	1.029	1.201	0.007	1.117	1.032	1.208	0.006
Gender				0.952				
Female		reference				NA		
Male	0.999	0.968	1.031	0.952				
Age(years)				<0.001				<0.001
< 65		reference				reference		
≥ 65	1.379	1.335	1.424	<0.001	1.273	1.231	1.315	<0.001
Marital status				<0.001				<0.001
Married		reference				reference		
Unmarried/NOS	1.216	1.178	1.256	<0.001	1.145	1.108	1.182	<0.001
Race				<0.001				0.003
White		reference				reference		
Non-white	1.076	1.035	1.119	<0.001	1.061	1.020	1.103	0.003
Primary tumor location				<0.001				0.676
Pancreas Head		reference				reference		

Pancreas Body/Tail	0.969	0.934	1.006	0.096	0.993	0.957	1.030	0.708
Pancreas Other	1.069	1.027	1.113	0.001	1.011	0.970	1.054	0.591
Pathologic grade				<0.001				<0.001
Grade I/II		reference				reference		
Grade III/IV	1.830	1.705	1.965	<0.001	1.854	1.726	1.991	<0.001
Unknown	1.795	1.698	1.897	<0.001	1.579	1.491	1.671	<0.001
Histologic type				0.019				0.103
Adenocarcinomas		reference				reference		
MCC/SRCC	1.107	1.017	1.206	0.019	1.073	0.986	1.169	0.103
T staging				<0.001				0.509
T0-3		reference				reference		
T4	1.045	1.001	1.091	0.043	1.012	0.969	1.057	0.583
Tx	1.187	1.143	1.233	<0.001	1.024	0.983	1.068	0.258
N staging				<0.001				0.010
N0		reference				reference		
N+	0.975	0.942	1.010	0.167	1.050	1.013	1.088	0.008
Nx	1.150	1.100	1.203	<0.001	1.055	1.006	1.106	0.028
Pancreatectomy				<0.001				<0.001
Yes		reference				reference		
No	4.074	3.592	4.620	<0.001	3.813	3.349	4.343	<0.001
Chemotherapy				<0.001				<0.001
Yes		reference				reference		
No	1.802	1.744	1.862	<0.001	1.917	1.853	1.984	<0.001
Bone metastasis				<0.001				<0.001
Yes		reference				reference		

No	0.797	0.748	0.849	<0.001	0.836	0.782	0.893	<0.001
Brain metastasis				<0.001				<0.001
Yes		reference				reference		
No	0.577	0.468	0.713	<0.001	0.671	0.541	0.833	<0.001
Lung metastasis				<0.001				<0.001
Yes		reference				reference		
No	0.724	0.694	0.755	<0.001	0.755	0.723	0.789	<0.001
PDAC lung metastasis								
Characteristics	Univariable analysis				Multivariable analysis			
	HR	95% CI lower	95% CI upper	*p*-value	HR	95% CI lower	95% CI upper	*p*-value
**Radiotherapy**				**0.165**				**0.641**
**Yes**		**reference**				**reference**		
**No**	**1.086**	**0.967**	**1.221**	**0.165**	**1.030**	**0.909**	**1.168**	**0.641**
Insurance				0.193				
Yes		reference				NA		
No/NOS	1.103	0.952	1.278	0.193				
Gender				0.002				<0.001
Female		reference				reference		
Male	1.100	1.036	1.169	0.002	1.123	1.055	1.196	<0.001
Age(years)				<0.001				<0.001
< 65		reference				reference		
≥ 65	1.176	1.105	1.253	<0.001	1.210	1.134	1.290	<0.001
Marital status				<0.001				<0.001
Married		reference				reference		
Unmarried/NOS	1.217	1.144	1.293	<0.001	1.137	1.067	1.211	<0.001

Race				0.112				0.167
White		reference				reference		
Non-white	1.063	0.986	1.146	0.112	1.055	0.978	1.138	0.167
Primary tumor location				<0.001				0.254
Pancreas Head		reference				reference		
Pancreas Body/Tail	1.096	1.019	1.179	0.014	1.065	0.988	1.147	0.099
Pancreas Other	1.183	1.096	1.277	<0.001	1.039	0.959	1.126	0.345
Pathologic grade				<0.001				<0.001
Grade I/II		reference				reference		
Grade III/IV	1.338	1.165	1.538	<0.001	1.328	1.155	1.527	<0.001
Unknown	1.234	1.112	1.370	<0.001	1.179	1.061	1.311	0.002
Histologic type				<0.001				0.273
Adenocarcinomas		reference				reference		
MCC/SRCC	0.808	0.718	0.908	<0.001	0.936	0.831	1.054	0.273
T staging				<0.001				0.508
T0-3		reference				reference		
T4	0.932	0.863	1.008	0.078	0.976	0.902	1.056	0.547
Tx	1.181	1.098	1.271	<0.001	1.032	0.953	1.118	0.434
N staging				<0.001				0.001
N0		reference				reference		
N+	1.027	0.961	1.097	0.433	1.083	1.012	1.159	0.021
Nx	1.242	1.138	1.355	<0.001	1.176	1.072	1.290	0.001
Pancreatectomy				<0.001				<0.001
Yes		reference				reference		
No	2.486	1.812	3.412	<0.001	2.277	1.649	3.143	<0.001

Chemotherapy				<0.001				<0.001
Yes		reference				reference		
No	2.173	2.038	2.317	<0.001	2.257	2.112	2.412	<0.001
Bone metastasis				<0.001				<0.001
Yes		reference				reference		
No	0.800	0.734	0.873	<0.001	0.813	0.741	0.891	<0.001
Brain metastasis				0.098				0.130
Yes		reference				reference		
No	0.818	0.645	1.038	0.098	0.825	0.643	1.059	0.130
Liver metastasis				<0.001				<0.001
Yes		reference				reference		
No	0.657	0.617	0.700	<0.001	0.645	0.604	0.689	<0.001
PDAC bone metastasis								
Characteristics	Univariable analysis				Multivariable analysis			
	HR	95% CI lower	95% CI upper	*p*-value	HR	95% CI lower	95% CI upper	*p*-value
**Radiotherapy**				**0.493**				**0.924**
**Yes**		**reference**				**reference**		
**No**	**1.040**	**0.929**	**1.165**	**0.493**	**0.994**	**0.886**	**1.117**	**0.924**
Insurance				0.805				
Yes		reference				NA		
No/NOS	1.030	0.815	1.301	0.805				
Gender				0.314				0.130
Female		reference				reference		
Male	1.054	0.951	1.168	0.314	1.086	0.976	1.209	0.130
Age(years)				0.001				0.006

< 65		reference				reference		
≥ 65	1.195	1.079	1.324	0.001	1.158	1.043	1.285	0.006
Marital status				0.004				0.045
Married		reference				reference		
Unmarried/NOS	1.164	1.051	1.289	0.004	1.116	1.002	1.242	0.045
Race				0.997				
White		reference				NA		
Non-white	1.000	0.882	1.134	0.997				
Primary tumor location				0.019				0.545
Pancreas Head		reference				reference		
Pancreas Body/Tail	1.039	0.915	1.180	0.554	1.042	0.916	1.186	0.532
Pancreas Other	1.188	1.043	1.353	0.010	1.080	0.942	1.240	0.270
Pathologic grade				<0.001				<0.001
Grade I/II		reference				reference		
Grade III/IV	1.803	1.423	2.283	<0.001	2.108	1.657	2.682	<0.001
Unknown	1.724	1.434	2.073	<0.001	1.764	1.460	2.132	<0.001
Histologic type				0.520				
Adenocarcinomas		reference				NA		
MCC/SRCC	1.075	0.863	1.340	0.520				
T staging				0.028				0.823
T0-3		reference				reference		
T4	0.968	0.842	1.113	0.648	1.001	0.870	1.152	0.991
Tx	1.150	1.025	1.291	0.018	1.040	0.916	1.180	0.547
N staging				0.035				0.175
N0		reference				reference		

N+	1.099	0.984	1.229	0.095	1.104	0.986	1.238	0.087
Nx	1.201	1.038	1.389	0.014	1.107	0.951	1.288	0.190
Pancreatectomy				0.002				0.010
Yes		reference				reference		
No	2.726	1.463	5.078	0.002	2.318	1.224	4.392	0.010
Chemotherapy				<0.001				<0.001
Yes		reference				reference		
No	1.902	1.709	2.117	<0.001	2.085	1.863	2.333	<0.001
Brain metastasis				0.132				0.160
Yes		reference				reference		
No	0.807	0.610	1.067	0.132	0.813	0.609	1.085	0.160
Liver metastasis				<0.001				<0.001
Yes		reference				reference		
No	0.820	0.736	0.913	<0.001	0.786	0.703	0.878	<0.001
Lung metastasis				<0.001				<0.001
Yes		reference				reference		
No	0.794	0.716	0.881	<0.001	0.783	0.703	0.872	<0.001
PDAC brain metastasis								
Characteristics	Univariable analysis				Multivariable analysis			
	HR	95% CI lower	95% CI upper	*p*-value	HR	95% CI lower	95% CI upper	*p*-value
**Radiotherapy**				**0.290**				**0.642**
**Yes**		**reference**				**reference**		
**No**	**1.192**	**0.861**	**1.652**	**0.290**	**1.082**	**0.777**	**1.505**	**0.642**
Insurance				0.778				
Yes		reference				NA		

No/NOS	0.901	0.437	1.859	0.778				
Gender				0.206				0.098
Female		reference				reference		
Male	1.236	0.890	1.718	0.206	1.328	0.949	1.858	0.098
Age(years)				0.007				0.001
< 65								
≥ 65	1.584	1.133	2.215	0.007	1.829	1.295	2.583	0.001
Marital status				0.367				
Married		reference				NA		
Unmarried/NOS	1.163	0.837	1.616	0.367				
Race				0.731				
White		reference				NA		
Non-white	1.071	0.723	1.588	0.731				
Primary tumor location				0.580				
Pancreas Head		reference				NA		
Pancreas Body/Tail	1.232	0.815	1.863	0.322				
Pancreas Other	1.196	0.788	1.814	0.401				
Pathologic grade				.466				
Grade I/II		reference				NA		
Grade III/IV	1.279	0.584	2.803	0.539				
Unknown	1.484	0.748	2.944	0.258				
Histologic type				0.670				
Adenocarcinomas		reference				NA		
MCC/SRCC	0.868	0.453	1.663	0.670				
T staging				0.872				

T0-3		reference				NA		
T4	0.868	0.509	1.480	0.604				
Tx	0.982	0.694	1.388	0.916				
N staging				0.219				0.023
N0		reference				NA		
N+	1.373	0.948	1.990	0.094	1.582	1.087	2.301	0.017
Nx	1.278	0.818	1.997	0.282	1.671	1.055	2.647	0.029
Pancreatectomy				0.518				
Yes		reference				NA		
No	1.916	0.267	13.762	0.518				
Chemotherapy				<0.001				
Yes		reference				reference		
No	2.991	2.089	4.283	<0.001	3.473	2.373	5.083	<0.001
Bone metastasis				1.000				
Yes		reference				NA		
No	1.000	0.709	1.411	1.000				
Liver metastasis				0.015				0.011
Yes		reference				reference		
No	0.658	0.470	0.921	0.015	0.642	0.456	0.904	0.011
Lung metastasis				0.710				
Yes		reference				NA		
No	1.064	0.767	1.477	0.710				

MCC: mucinous cell carcinoma; SRCC: signet ring cell carcinoma.

Table S2 Characteristics of metastatic PDAC before and after PSM

PDAC liver metastasis										
Characteristics	Before PSM					After PSM				
	Non-radiotherapy (n=16993)		Radiotherapy (n=829)		*p*-value	Non-radiotherapy (n=561)		Radiotherapy (n=561)		*p*-value
	N	%	N	%		N	%	N	%	
Insurance					0.121					0.585
Yes	16280	95.80%	785	94.69%		544	96.97%	547	97.50%	
No/NOS	713	4.20%	44	5.31%		17	3.03%	14	2.50%	
Gender					0.343					1.000
Female	7747	45.59%	364	43.91%		248	44.21%	248	44.21%	
Male	9246	54.41%	465	56.09%		313	55.79%	313	55.79%	
Age(years)					<0.001					0.676
< 65	7135	41.99%	421	50.78%		273	48.66%	266	47.42%	
≥ 65	9858	58.01%	408	49.22%		288	51.34%	295	52.58%	
Marital status					0.131					0.806
Married	9695	57.05%	495	59.71%		345	61.50%	349	62.21%	
Unmarried/NOS	7298	42.95%	334	40.29%		216	38.50%	212	37.79%	
Race					0.913					0.815
White	13502	79.46%	660	79.61%		460	82.00%	463	82.53%	
Non-white	3491	20.54%	169	20.39%		101	18.00%	98	17.47%	
Primary tumor location					0.130					0.475
Pancreas Head	6132	36.09%	329	39.69%		234	41.71%	244	43.49%	
Pancreas Body/Tail	6347	37.35%	285	34.38%		189	33.69%	188	33.51%	
Pancreas Other	4514	26.56%	215	25.93%		138	24.60%	129	22.99%	

Pathologic grade					0.069					0.849
Grade I/II	1814	10.67%	90	10.86%		52	9.27%	51	9.09%	
Grade III/IV	1799	10.59%	120	14.48%		67	11.94%	65	11.59%	
Unknown	13380	78.74%	619	74.67%		442	78.79%	445	79.32%	
Histologic type					0.171					1.000
Adenocarcinomas	16425	96.66%	794	95.78%		555	98.93%	555	98.93%	
MCC/SRCC	568	3.34%	35	4.22%		6	1.07%	6	1.07%	
T staging					0.950					0.842
T0-3	10081	59.32%	471	56.82%		352	62.75%	352	62.75%	
T4	2949	17.35%	187	22.56%		119	21.21%	124	22.10%	
Tx	3963	23.32%	171	20.63%		90	16.04%	85	15.15%	
N staging					0.171					0.396
N0	8772	51.62%	384	46.32%		281	50.09%	289	51.52%	
N+	5571	32.78%	330	39.81%		217	38.68%	220	39.22%	
Nx	2650	15.59%	115	13.87%		63	11.23%	52	9.27%	
Pancreatectomy					0.031					0.851
Yes	479	2.82%	34	4.10%		15	2.67%	14	2.50%	
No	16514	97.18%	795	95.90%		546	97.33%	547	97.50%	
Chemotherapy					<0.001					0.890
Yes	10530	61.97%	597	72.01%		421	75.04%	423	75.40%	
No	6463	38.03%	232	27.99%		140	24.96%	138	24.60%	
Bone metastasis					<0.001					1.000
Yes	845	4.97%	266	32.09%		92	16.40%	92	16.40%	
No	16148	95.03%	563	67.91%		469	83.60%	469	83.60%	
Brain metastasis					<0.001					1.000

Yes	48	0.28%	42	5.07%		1	0.18%	1	0.18%	
No	16945	99.72%	787	94.93%		560	99.82%	560	99.82%	
Lung metastasis					<0.001					0.715
Yes	2667	15.69%	169	20.39%		66	11.76%	70	12.48%	
No	14326	84.31%	660	79.61%		495	88.24%	491	87.52%	
PDAC lung metastasis										
Characteristics	Before PSM					After PSM				
	Non-radiotherapy (n=4379)		Radiotherapy (n=338)		*p*-value	Non-radiotherapy (n=162)		Radiotherapy (n=162)		*p*-value
	N	%	N	%		N	%	N	%	
Insurance					0.990					0.177
Yes	4197	95.84%	324	95.86%		161	99.38%	158	97.53%	
No/NOS	182	4.16%	14	4.14%		1	0.62%	4	2.47%	
Gender					0.068					0.656
Female	2221	50.72%	154	45.56%		70	43.21%	74	45.68%	
Male	2158	49.28%	184	54.44%		92	56.79%	88	54.32%	
Age(years)					0.003					0.910
< 65	1592	36.36%	150	44.38%		67	41.36%	66	40.74%	
≥ 65	2787	63.64%	188	55.62%		95	58.64%	96	59.26%	
Marital status					0.423					0.823
Married	2482	56.68%	184	54.44%		90	55.56%	92	56.79%	
Unmarried/NOS	1897	43.32%	154	45.56%		72	44.44%	70	43.21%	
Race					0.390					0.667
White	3504	80.02%	277	81.95%		134	82.72%	131	80.86%	
Non-white	875	19.98%	61	18.05%		28	17.28%	31	19.14%	
Primary tumor location					0.805					0.835

Pancreas Head	1438	32.84%	109	32.25%		58	35.80%	58	35.80%	
Pancreas Body/Tail	1629	37.20%	126	37.28%		57	35.19%	60	37.04%	
Pancreas Other	1312	29.96%	103	30.47%		47	29.01%	44	27.16%	
Pathologic grade					0.100					0.257
Grade I/II	412	9.41%	38	11.24%		19	11.73%	14	8.64%	
Grade III/IV	410	9.36%	39	11.54%		13	8.02%	10	6.17%	
Unknown	3557	81.23%	261	77.22%		130	80.25%	138	85.19%	
Histologic type					0.173					0.792
Adenocarcinomas	4065	92.83%	307	90.83%		154	95.06%	155	95.68%	
MCC/SRCC	314	7.17%	31	9.17%		8	4.94%	7	4.32%	
T staging					0.587					0.236
T0-3	2380	54.35%	177	52.37%		87	53.70%	95	58.64%	
T4	936	21.37%	77	22.78%		38	23.46%	39	24.07%	
Tx	1063	24.27%	84	24.85%		37	22.84%	28	17.28%	
N staging					0.231					0.861
N0	1921	43.87%	135	39.94%		70	43.21%	72	44.44%	
N+	1749	39.94%	145	42.90%		80	49.38%	74	45.68%	
Nx	709	16.19%	58	17.16%		12	7.41%	16	9.88%	
Pancreatectomy					0.238					0.318
Yes	47	1.07%	6	1.78%		1	0.62%	0	0.00%	
No	4332	98.93%	332	98.22%		161	99.38%	162	100.00%	
Chemotherapy					<0.001					0.691
Yes	2719	62.09%	251	74.26%		124	76.54%	127	78.40%	
No	1660	37.91%	87	25.74%		38	23.46%	35	21.60%	
Bone metastasis					<0.001					0.808

Yes	474	10.82%	165	48.82%		47	29.01%	49	30.25%	
No	3905	89.18%	173	51.18%		115	70.99%	113	69.75%	
Brain metastasis					<0.001					0.318
Yes	34	0.78%	41	12.13%		1	0.62%	0	0.00%	
No	4345	99.22%	297	87.87%		161	99.38%	162	100.00%	
Liver metastasis					<0.001					0.912
Yes	2667	60.90%	169	50.00%		83	51.23%	82	50.62%	
No	1712	39.10%	169	50.00%		79	48.77%	80	49.38%	
PDAC bone metastasis										
Characteristics	Before PSM					After PSM				
	Non-radiotherapy (n=1209)		Radiotherapy (n=457)		*p*-value	Non-radiotherapy (n=146)		Radiotherapy (n=146)		*p*-value
	N	%	N	%		N	%	N	%	
Insurance					0.666					0.653
Yes	1150	95.12%	437	95.62%		144	98.63%	143	97.95%	
No/NOS	59	4.88%	20	4.38%		2	1.37%	3	2.05%	
Gender					0.424					0.902
Female	513	42.43%	184	40.26%		50	34.25%	49	33.56%	
Male	696	57.57%	273	59.74%		96	65.75%	97	66.44%	
Age(years)					0.211					0.905
< 65	517	42.76%	211	46.17%		57	39.04%	58	39.73%	
≥ 65	692	57.24%	246	53.83%		89	60.96%	88	60.27%	
Marital status					0.391					0.811
Married	708	58.56%	257	56.24%		91	62.33%	89	60.96%	
Unmarried/NOS	501	41.44%	200	43.76%		55	37.67%	57	39.04%	
Race					0.029					0.659

White	947	78.33%	380	83.15%		136	93.15%	134	91.78%	
Non-white	262	21.67%	77	16.85%		10	6.85%	12	8.22%	
Primary tumor location					0.055					0.871
Pancreas Head	340	28.12%	106	23.19%		30	20.55%	26	17.81%	
Pancreas Body/Tail	464	38.38%	183	40.04%		64	43.84%	70	47.95%	
Pancreas Other	405	33.50%	168	36.76%		52	35.62%	50	34.25%	
Pathologic grade					0.677					0.889
Grade I/II	117	9.68%	42	9.19%		6	4.11%	5	3.42%	
Grade III/IV	111	9.18%	53	11.60%		5	3.42%	6	4.11%	
Unknown	981	81.14%	362	79.21%		135	92.47%	135	92.47%	
Histologic type					0.329					0.411
Adenocarcinomas	1147	94.87%	428	93.65%		142	97.26%	144	98.63%	
MCC/SRCC	62	5.13%	29	6.35%		4	2.74%	2	1.37%	
T staging					0.146					0.895
T0-3	647	53.52%	229	50.11%		84	57.53%	87	59.59%	
T4	214	17.70%	80	17.51%		21	14.38%	17	11.64%	
Tx	348	28.78%	148	32.39%		41	28.08%	42	28.77%	
N staging					0.535					0.730
N0	503	41.60%	199	43.54%		67	45.89%	70	47.95%	
N+	516	42.68%	166	36.32%		62	42.47%	60	41.10%	
Nx	190	15.72%	92	20.13%		17	11.64%	16	10.96%	
Pancreatectomy					0.465					1.000
Yes	11	0.91%	6	1.31%		0	0.00%	0	0.00%	
No	1198	99.09%	451	98.69%		146	100.00%	146	100.00%	
Chemotherapy					0.099					0.698

Yes	746	61.70%	302	66.08%		106	72.60%	103	70.55%	
No	463	38.30%	155	33.92%		40	27.40%	43	29.45%	
Brain metastasis					<0.001					1.000
Yes	23	1.90%	31	6.78%		0	0.00%	0	0.00%	
No	1186	98.10%	426	93.22%		146	100.00%	146	100.00%	
Liver metastasis					<0.001					0.800
Yes	845	69.89%	266	58.21%		103	70.55%	101	69.18%	
No	364	30.11%	191	41.79%		43	29.45%	45	30.82%	
Lung metastasis					0.246					0.902
Yes	474	39.21%	165	36.11%		49	33.56%	48	32.88%	
No	735	60.79%	292	63.89%		97	66.44%	98	67.12%	
PDAC brain metastasis										
Characteristics	Before PSM					After PSM				
	Non-radiotherapy (n=81)		Radiotherapy (n=78)		*p*-value	Non-radiotherapy (n=21)		Radiotherapy (n=21)		*p*-value
	N	%	N	%		N	%	N	%	
Insurance					0.505					1.000
Yes	76	93.83%	75	96.15%		21	100.00%	21	100.00%	
No/NOS	5	6.17%	3	3.85%		0	0.00%	0	0.00%	
Gender					0.703					0.358
Female	37	45.68%	38	48.72%		7	33.33%	10	47.62%	
Male	44	54.32%	40	51.28%		14	66.67%	11	52.38%	
Age(years)					0.136					0.760
< 65	34	41.98%	42	53.85%		8	38.10%	9	42.86%	
≥ 65	47	58.02%	36	46.15%		13	61.90%	12	57.14%	
Marital status					0.588					0.755

Married	44	54.32%	39	50.00%		13	61.90%	14	66.67%	
Unmarried/NOS	37	45.68%	39	50.00%		8	38.10%	7	33.33%	
Race					0.942					0.444
White	64	79.01%	62	79.49%		18	85.71%	16	76.19%	
Non-white	17	20.99%	16	20.51%		3	14.29%	5	23.81%	
Primary tumor location					0.861					1.000
Pancreas Head	22	27.16%	23	29.49%		6	28.57%	5	23.81%	
Pancreas Body/Tail	30	37.04%	27	34.62%		6	28.57%	8	38.10%	
Pancreas Other	29	35.80%	28	35.90%		9	42.86%	8	38.10%	
Pathologic grade					0.598					0.633
Grade I/II	4	4.94%	6	7.69%		1	4.76%	3	14.29%	
Grade III/IV	12	14.81%	11	14.10%		3	14.29%	1	4.76%	
Unknown	65	80.25%	61	78.21%		17	80.95%	17	80.95%	
Histologic type					0.708					0.081
Adenocarcinomas	76	93.83%	72	92.31%		20	95.24%	16	76.19%	
MCC/SRCC	5	6.17%	6	7.69%		1	4.76%	5	23.81%	
T staging					0.445					0.619
T0-3	33	40.74%	38	48.72%		9	42.86%	11	52.38%	
T4	12	14.81%	8	10.26%		4	19.05%	3	14.29%	
Tx	36	44.44%	32	41.03%		8	38.10%	7	33.33%	
N staging					0.767					1.000
N0	38	46.91%	35	44.87%		9	42.86%	9	42.86%	
N+	26	32.10%	31	39.74%		8	38.10%	8	38.10%	
Nx	17	20.99%	12	15.38%		4	19.05%	4	19.05%	
Pancreatectomy					0.328					1.000

Yes	1	1.23%	0	0.00%		0	0.00%	0	0.00%	
No	80	98.77%	78	100.00%		21	100.00%	21	100.00%	
Chemotherapy					0.091					0.358
Yes	39	48.15%	48	61.54%		14	66.67%	11	52.38%	
No	42	51.85%	30	38.46%		7	33.33%	10	47.62%	
Bone metastasis					0.133					0.524
Yes	23	28.40%	31	39.74%		6	28.57%	8	38.10%	
No	58	71.60%	47	60.26%		15	71.43%	13	61.90%	
Liver metastasis					0.494					1.000
Yes	48	59.26%	42	53.85%		11	52.38%	11	52.38%	
No	33	40.74%	36	46.15%		10	47.62%	10	47.62%	
Lung metastasis					0.183					0.358
Yes	34	41.98%	41	52.56%		10	47.62%	7	33.33%	
No	47	58.02%	37	47.44%		11	52.38%	14	66.67%	
MCC: mucinous cell carcinoma; SRCC: signet ring cell carcinoma.										

Table S3 Univariable and multivariable Cox regression model in PDAC-liver-metastasis patients with or without LBB

PDAC-liver-metastasis with LBB								
Characteristics	Univariable analysis				Multivariable analysis			
	HR	95% CI lower	95% CI upper	*p*-value	HR	95% CI lower	95% CI upper	*p*-value
**Radiotherapy**				0.022				0.557

**Yes**		reference				reference		
**No**	1.145	1.020	1.285	0.022	1.038	0.917	1.173	0.557
Insurance				0.303				
Yes		reference				NA		
No/NOS	1.091	0.924	1.289	0.303				
Gender				0.504				
Female		reference				NA		
Male	1.024	0.955	1.097	0.504				
Age(years)				<0.001				<0.001
< 65		reference				reference		
≥ 65	1.223	1.140	1.312	<0.001	1.149	1.070	1.234	<0.001
Marital status				<0.001				0.001
Married		reference				reference		
Unmarried/NOS	1.224	1.142	1.313	<0.001	1.125	1.048	1.207	0.001
Race				0.053				
White		reference				NA		
Non-white	1.088	0.999	1.184	0.053				
Primary tumor location				0.070				
Pancreas Head		reference				NA		
Pancreas Body/Tail	0.990	0.908	1.078	0.810				
Pancreas Other	1.083	0.991	1.185	0.080				
Pathologic grade				<0.001				<0.001
Grade I/II		reference				reference		
Grade III/IV	1.530	1.301	1.800	<0.001	1.619	1.375	1.907	<0.001
Unknown	1.445	1.271	1.644	<0.001	1.432	1.258	1.631	<0.001

Histologic type				0.850				
Adenocarcinomas		reference				NA		
MCC/SRCC	0.984	0.828	1.169	0.850				
T staging				0.008				0.394
T0-3		reference				reference		
T4	0.947	0.864	1.037	0.238	0.948	0.864	1.039	0.251
Tx	1.105	1.018	1.199	0.017	1.016	0.933	1.108	0.711
N staging				0.001				0.011
N0		reference				reference		
N+	1.007	0.933	1.086	0.862	1.092	1.011	1.179	0.025
Nx	1.203	1.088	1.331	<0.001	1.156	1.040	1.284	0.007
Pancreatectomy				<0.001				<0.001
Yes		reference				reference		
No	3.086	1.750	5.442	<0.001	2.954	1.668	5.231	<0.001
Chemotherapy				<0.001				<0.001
Yes		reference				reference		
No	2.265	2.104	2.439	<0.001	2.258	2.092	2.436	<0.001
Bone metastasis				0.282				
Yes		reference				NA		
No	1.042	0.967	1.122	0.282				
Brain metastasis				0.004				0.002
Yes		reference				reference		
No	0.733	0.593	0.908	0.004	0.711	0.570	0.887	0.002
Lung metastasis				0.001				<0.001
Yes		reference				reference		

No	0.859	0.787	0.937	0.001	0.847	0.774	0.928	<0.001
PDAC-liver-metastasis without LBB								
Characteristics	Univariable analysis				Multivariable analysis			
	HR	95% CI lower	95% CI upper	*p*-value	HR	95% CI lower	95% CI upper	*p*-value
**Radiotherapy**				<0.001				0.001
**Yes**		reference				reference		
**No**	1.290	1.169	1.425	<0.001	1.187	1.075	1.311	0.001
Insurance				0.016				0.005
Yes		reference				reference		
No/NOS	1.114	1.020	1.215	0.016	1.136	1.039	1.241	0.005
Gender				0.759				
Female		reference				NA		
Male	0.994	0.960	1.030	0.759				
Age(years)				<0.001				<0.001
< 65		reference				reference		
≥ 65	1.419	1.369	1.471	<0.001	1.304	1.257	1.353	<0.001
Marital status				<0.001				<0.001
Married		reference				reference		
Unmarried/NOS	1.217	1.175	1.261	<0.001	1.149	1.108	1.191	<0.001
Race				0.001				0.013
White		reference				reference		
Non-white	1.074	1.028	1.121	0.001	1.057	1.012	1.105	0.013
Primary tumor location				<0.001				0.670
Pancreas Head		reference				reference		
Pancreas Body/Tail	0.944	0.906	0.983	0.006	0.983	0.943	1.024	0.411

Pancreas Other	1.035	0.990	1.083	0.129	0.999	0.954	1.047	0.970
Pathologic grade				<0.001				<0.001
Grade I/II		reference				reference		
Grade III/IV	1.882	1.740	2.036	<0.001	1.906	1.761	2.064	<0.001
Unknown	1.851	1.741	1.969	<0.001	1.610	1.511	1.715	<0.001
Histologic type				0.016				0.061
Adenocarcinomas		reference				reference		
MCC/SRCC	1.128	1.023	1.244	0.016	1.098	0.996	1.211	0.061
T staging				<0.001				0.330
T0-3		reference				reference		
T4	1.054	1.004	1.107	0.033	1.029	0.980	1.081	0.252
Tx	1.190	1.140	1.242	<0.001	1.030	0.982	1.079	0.227
N staging				<0.001				0.116
N0		reference				reference		
N+	0.937	0.901	0.976	0.002	1.042	1.000	1.085	0.050
Nx	1.126	1.071	1.184	<0.001	1.032	0.978	1.089	0.245
Pancreatectomy				<0.001				<0.001
Yes		reference				reference		
No	4.003	3.517	4.555	<0.001	3.791	3.315	4.335	<0.001
Chemotherapy				<0.001				<0.001
Yes		reference				reference		
No	1.737	1.674	1.802	<0.001	1.842	1.773	1.913	<0.001
MCC: mucinous cell carcinoma; SRCC: signet ring cell carcinoma.								

Table S4 Characteristics of PDAC-liver-metastasis with or without LBB before and after PSM

PDAC-liver-metastasis with LBB										
Characteristics	Before PSM					After PSM				
	Non-radiotherapy (n=3200)		Radiotherapy (n=350)		*p*-value	Non-radiotherapy (n=136)		Radiotherapy (n=136)		*p*-value
	N	%	N	%		N	%	N	%	
Insurance					0.800					0.411
Yes	3063	95.72%	334	95.43%		134	98.53%	132	97.06%	
No/NOS	137	4.28%	16	4.57%		2	1.47%	4	2.94%	
Gender					0.028					0.465
Female	1487	46.47%	141	40.29%		62	45.59%	56	41.18%	
Male	1713	53.53%	209	59.71%		74	54.41%	80	58.82%	
Age(years)					0.001					0.807
< 65	1310	40.94%	176	50.29%		57	41.91%	59	43.38%	
≥ 65	1890	59.06%	174	49.71%		79	58.09%	77	56.62%	
Marital status					0.662					1.000
Married	1831	57.22%	196	56.00%		83	61.03%	83	61.03%	
Unmarried/NOS	1369	42.78%	154	44.00%		53	38.97%	53	38.97%	
Race					0.398					0.849
White	2535	79.22%	284	81.14%		120	88.24%	121	88.97%	
Non-white	665	20.78%	66	18.86%		16	11.76%	15	11.03%	
Primary tumor location					0.495					0.681
Pancreas Head	884	27.63%	92	26.29%		31	22.79%	31	22.79%	
Pancreas Body/Tail	1298	40.56%	141	40.29%		59	43.38%	64	47.06%	
Pancreas Other	1018	31.81%	117	33.43%		46	33.82%	41	30.15%	
Pathologic grade					0.036					0.711

Grade I/II	262	8.19%	38	10.86%		8	5.88%	7	5.15%	
Grade III/IV	326	10.19%	42	12.00%		6	4.41%	5	3.68%	
Unknown	2612	81.63%	270	77.14%		122	89.71%	124	91.18%	
Histologic type					0.082					0.157
Adenocarcinomas	3071	95.97%	329	94.00%		134	98.53%	136	100.00%	
MCC/SRCC	129	4.03%	21	6.00%		2	1.47%	0	0.00%	
T staging					0.056					0.942
T0-3	1737	54.28%	174	49.71%		83	61.03%	80	58.82%	
T4	632	19.75%	69	19.71%		21	15.44%	28	20.59%	
Tx	831	25.97%	107	30.57%		32	23.53%	28	20.59%	
N staging					0.014					0.925
N0	1354	42.31%	127	36.29%		58	42.65%	57	41.91%	
N+	1341	41.91%	154	44.00%		65	47.79%	66	48.53%	
Nx	505	15.78%	69	19.71%		13	9.56%	13	9.56%	
Pancreatectomy					0.031					1.000
Yes	16	0.50%	5	1.43%		0	0.00%	0	0.00%	
No	3184	99.50%	345	98.57%		136	100.00%	136	100.00%	
Chemotherapy					0.017					1.000
Yes	1986	62.06%	240	68.57%		99	72.79%	99	72.79%	
No	1214	37.94%	110	31.43%		37	27.21%	37	27.21%	
Bone metastasis					<0.001					0.896
Yes	845	26.41%	266	76.00%		95	69.85%	94	69.12%	
No	2355	73.59%	84	24.00%		41	30.15%	42	30.88%	
Brain metastasis					<0.001					0.563
Yes	48	1.50%	42	12.00%		1	0.74%	2	1.47%	

No	3152	98.50%	308	88.00%		135	99.26%	134	98.53%	
Lung metastasis					<0.001					0.904
Yes	2667	83.34%	169	48.29%		73	53.68%	74	54.41%	
No	533	16.66%	181	51.71%		63	46.32%	62	45.59%	
PDAC-liver-metastasis without LBB										
Characteristics	Before PSM					After PSM				
	Non-radiotherapy (n=13793)		Radiotherapy (n=479)		*p*-value	Non-radiotherapy (n=426)		Radiotherapy (n=426)		*p*-value
	N	%	N	%		N	%	N	%	
Insurance					0.074					1.000
Yes	13217	95.82%	451	94.15%		415	97.42%	415	97.42%	
No/NOS	576	4.18%	28	5.85%		11	2.58%	11	2.58%	
Gender					0.613					0.945
Female	6260	45.39%	223	46.56%		193	45.31%	192	45.07%	
Male	7533	54.61%	256	53.44%		233	54.69%	234	54.93%	
Age(years)					<0.001					0.837
< 65	5825	42.23%	245	51.15%		212	49.77%	209	49.06%	
≥ 65	7968	57.77%	234	48.85%		214	50.23%	217	50.94%	
Marital status					0.019					0.888
Married	7864	57.01%	299	62.42%		266	62.44%	268	62.91%	
Unmarried/NOS	5929	42.99%	180	37.58%		160	37.56%	158	37.09%	
Race					0.589					0.930
White	10967	79.51%	376	78.50%		346	81.22%	345	80.99%	
Non-white	2826	20.49%	103	21.50%		80	18.78%	81	19.01%	
Primary tumor location					<0.001					0.930
Pancreas Head	5248	38.05%	237	49.48%		209	49.06%	211	49.53%	

Pancreas Body/Tail	5049	36.61%	144	30.06%		130	30.52%	128	30.05%	
Pancreas Other	3496	25.35%	98	20.46%		87	20.42%	87	20.42%	
Pathologic grade					0.121					1.000
Grade I/II	1552	11.25%	52	10.86%		42	9.86%	42	9.86%	
Grade III/IV	1473	10.68%	78	16.28%		59	13.85%	59	13.85%	
Unknown	10768	78.07%	349	72.86%		325	76.29%	325	76.29%	
Histologic type					0.750					0.402
Adenocarcinomas	13354	96.82%	465	97.08%		418	98.12%	421	98.83%	
MCC/SRCC	439	3.18%	14	2.92%		8	1.88%	5	1.17%	
T staging					0.005					0.740
T0-3	8344	60.49%	297	62.00%		268	62.91%	272	63.85%	
T4	2317	16.80%	118	24.63%		99	23.24%	98	23.00%	
Tx	3132	22.71%	64	13.36%		59	13.85%	56	13.15%	
N staging					0.089					0.798
N0	7418	53.78%	257	53.65%		230	53.99%	232	54.46%	
N+	4230	30.67%	176	36.74%		152	35.68%	153	35.92%	
Nx	2145	15.55%	46	9.60%		44	10.33%	41	9.62%	
Pancreatectomy					0.001					0.850
Yes	463	3.36%	30	6.26%		15	3.52%	14	3.29%	
No	13330	96.64%	449	93.74%		411	96.48%	412	96.71%	
Chemotherapy					<0.001					0.937
Yes	8544	61.94%	357	74.53%		322	75.59%	321	75.35%	
No	5249	38.06%	122	25.47%		104	24.41%	105	24.65%	

MCC: mucinous cell carcinoma; SRCC: signet ring cell carcinoma.
